# Differential relationship between decreased muscle oxygenation and blood pressure recovery during supraventricular and ventricular tachycardia

**DOI:** 10.1038/s41598-023-42908-2

**Published:** 2023-09-23

**Authors:** Kei Ishii, Takeshi Machino, Yasuhiro Hatori, Jongseong Gwak, Tsubasa Izaki, Hidehiko Komine

**Affiliations:** 1 Human Informatics and Interaction Research Institute, National Institute of Advanced Industrial Science and Technology, 1-1-1 Higashi, Tsukuba, Ibaraki 305-8566 Japan; 2 Department of Cardiology, University of Tsukuba, Tsukuba, Ibaraki Japan; 3 Research Institute of Electrical Communication, Tohoku University, Sendai, Miyagi Japan; 4 Department of Computer Science, Takushoku University, Hachioji, Tokyo Japan; 5 School of Economics and Management, Kochi University of Technology, Kochi, Kochi Japan

**Keywords:** Blood flow, Arrhythmias

## Abstract

Vasoconstriction during tachyarrhythmia contributes to maintenance of arterial pressure (AP) by decreasing peripheral blood flow. This cross-sectional observational study aimed to ascertain whether the relationship between peripheral blood flow and AP recovery occurs during both paroxysmal supraventricular (PSVT, n = 19) and ventricular tachycardias (VT, n = 17). Peripheral blood flow was evaluated using forearm tissue oxygen index (TOI), and mean AP (MAP) was measured using a catheter inserted in the brachial or femoral artery during an electrophysiological study. PSVT and VT rapidly decreased MAP with a comparable heart rate (*P* = 0.194). MAP recovered to the baseline level at 40 s from PSVT onset, but not VT. The forearm TOI decreased during both tachyarrhythmias (*P* ≤ 0.029). The TOI response was correlated with MAP_recovery_ (i.e., MAP recovery from the initial rapid decrease) at 20–60 s from PSVT onset (*r* = -– 0.652 to – 0.814, *P* ≤ 0.0298); however, this association was not observed during VT. These findings persisted even after excluding patients who had taken vasoactive drugs. Thus, restricting peripheral blood flow was associated with MAP recovery during PSVT, but not VT. This indicates that AP recovery depends on the type of tachyarrhythmia: different cardiac output and/or vasoconstriction ability during tachyarrhythmia.

## Introduction

Cardiac tachyarrhythmias are characterised by a rapid decrease in arterial pressure (AP), which depends on cardiac output (CO) and peripheral vasoconstriction primarily caused by arterial baroreflex-mediated activation of the sympathetic nervous system^1–4^. This hypotension can lead to cerebral hypoperfusion, which may result in a temporary loss of consciousness, also known as syncope. To prevent the onset of syncope in clinical and daily situations, understanding AP recovery during tachyarrhythmia is crucial.

The challenge of recovering from AP during tachyarrhythmia varies depending on the type of tachyarrhythmia. For example, stroke volume (SV) and CO are lower during ventricular tachycardia (VT) than during paroxysmal supraventricular tachycardia (PSVT), owing to the uncoordinated contraction of the atria and ventricles; this lower SV and CO persist even when the arrhythmia rate and heart function are similar between PSVT and VT^5–8^. The low CO during VT results in hypotension and insufficient blood supply to the peripheral vasculature. When the blood supply is lowered beyond a certain threshold, peripheral vasoconstriction would be unable to effectively increase peripheral vascular resistance, thereby failing to increase AP. Therefore, recovering AP during VT would be theoretically more difficult due to lower blood circulation.

Another important aspect is the ability to induce peripheral vasoconstriction during tachyarrhythmia. Left ventricular dysfunction and heart failure, which are common in patients with VT^9^, are associated with impaired baroreflex control of sympathetic nerve activity in response to AP changes (by < 20 mmHg), augmented sympathetic activity, and decreased vascular response to sympathetic stimulation^10–15^. These findings suggest that (1) the ability to evoke peripheral vasoconstriction in response to hypotension may be diminished in patients with VT and heart dysfunction and (2) measuring peripheral blood flow, but not sympathetic nerve activity, during tachyarrhythmia is a better way to assess the ability to evoke peripheral vasoconstriction in patients.

Thus, we hypothesised that the relationship between the peripheral blood flow response and AP recovery during tachyarrhythmia may differ between PSVT and VT, such that a decrease in peripheral blood flow following vasoconstriction would contribute to AP recovery during PSVT, but would have little effect on AP recovery during VT. To the best of our knowledge, this relationship during actual tachyarrhythmias has yet to be verified.

This cross-sectional observational study aimed to ascertain the relationship between peripheral blood flow and AP recovery during actual PSVT and VT occurred in electrophysiological study (EPS). The peripheral blood flow response to tachyarrhythmia was estimated by forearm oxygenation measured using near-infrared spectroscopy (NIRS). Among NIRS-derived variables (tissue oxygen index [TOI] and relative concentrations of oxygenated [Oxy-Hb] and deoxygenated [Deoxy-Hb] haemoglobin), TOI and Oxy-Hb in resting skeletal muscles are regarded as indices of regional tissue blood flow responses^16–19^. In particular, TOI appears to be more useful than Oxy-Hb for monitoring tissue oxygenation, given its lower sensitivity to changes in skin circulation^20^. To understand the relationship between peripheral blood flow and AP recovery during tachyarrhythmia in clinical and daily situations, we examined the AP and forearm TOI responses to PSVT and VT in patients after they had taken vasoactive drugs (if they regularly took them). Then, we narrowed down the possible factors contributing to this relationship by excluding the data of some confounding variables.

## Results

### Clinical characteristics

Fifty-six patients who were scheduled for EPS consented to participate. Of the 56 participants during EPS, 10 underwent implantable cardioverter defibrillator implantation under sedation, eight experienced only short-duration tachyarrhythmia (< 15 s) or a minor initial change (-10–10 mmHg) of mean AP (MAP) immediately after the onset of tachyarrhythmia, one experienced non-target arrhythmia (i.e., atrial fibrillation/tachycardia), one had no data on AP values owing to malfunction of the instruments. In the remaining 36 patients with PSVT (n = 19) or VT (n = 17), accepted tachyarrhythmias (> 15 s; the initial MAP changes < -10 mmHg or > 10 mmHg) were analysed in this study. The clinical characteristics of the patients are summarised in Table 1. Echocardiography demonstrated that SV was similar in patients with PSVT and VT, whereas heart function was diminished in patients with VT. Patients with VT had a higher prevalence of structural heart disease (*P* < 0.001) and chronic kidney disease (*P* = 0.04) than those with PSVT. One patient with PSVT and three patients with VT were diagnosed with heart failure.

**Table 1:** Clinical characteristics of the study population.

	PSVT (n = 19)	VT (n = 17)	*P*-value
Age (yrs)	53 ± 16	59 ± 18	0.254
Male/female sex	10/9	14/3	0.576
LVEF (%)	67 ± 6	41 ± 17	< 0.001*
LVEDV (mL)	86 ± 29	160 ± 54	< 0.001*
SV (mL)	58 ± 20	58 ± 16	0.975
E/e’	6.9 ± 2.1	11.7 ± 4.6	0.004*
Structural heart disease [n (%)]	1 (5)	14 (82)	< 0.001*
ICM (n)	1	6	
DCM (n)	0	3	
HCM (n)	0	0	
ARVC (n)	0	1	
Sarcoidosis (n)	0	3	
Brugada syndrome (n)	0	1	
Ebstein anomaly (n)	0	1	
N/A	18	3	
Hypertension [n (%)]	5 (26)	7 (41)	0.483
Diabetes [n (%)]	1 (5)	4 (24)	0.167
Chronic kidney disease [n (%)]	0 (0)	4 (24)	0.04*
Vasoactive drugs [n (%)]	6 (32)	14 (82)	0.003*
α1 and β1 receptor blockers	1	8	
Angiotensin II receptor blocker	3	10	
Aldosterone receptor blocker	0	9	
Vasopressin receptor blocker	0	2	
Calcium channel blocker	5	3	
N/A	13	3	

Values are presented as mean ± SD or n (%).

*ARVC* arrhythmogenic right ventricular cardiomyopathy, *DCM* dilated cardiomyopathy, *E*/e’ early diastolic transmitral velocity/early diastolic mitral annular tissue velocity, *HCM* hypertrophic cardiomyopathy, *ICM* ischemic cardiomyopathy, *LVEDV* left ventricular end-diastolic volume, *LVEF* left ventricular ejection fraction, *N/A* not applicable, *PSVT* paroxysmal supraventricular tachycardia, *SV* stroke volume, *VT* ventricular tachycardia.

*Significant difference (*P* < 0.05) between the PSVT and VT groups.

### Cardiovascular responses to actual PSVT and VT

**Figure 1:**
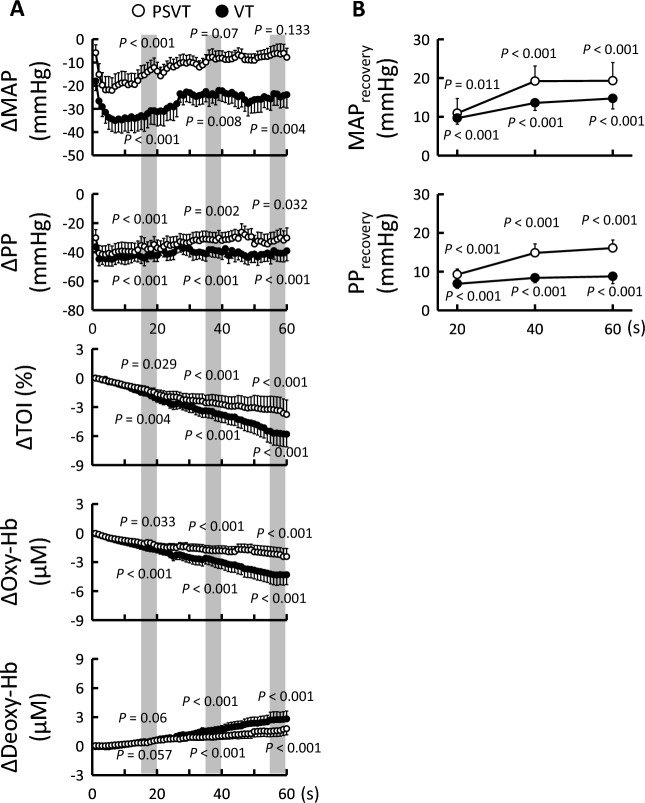
Time course of average cardiovascular responses during paroxysmal supraventricular tachycardia (PSVT, ○) and ventricular tachycardia (VT, ●). (**A)** The hemodynamic changes from baseline values were aligned at the onset of arrhythmia and averaged every 1 s. Grey areas indicate three time periods (16–20, 36–40, and 56–60 s of arrhythmia). The average values during each period were compared to baseline values and each *P*-value is described. (**B)** MAP_recovery_ or PP_recovery_ was calculated using the recovery of MAP or PP from the initial change, respectively. Because this study evaluated actual tachyarrhythmia, the arrhythmia duration could not be controlled; therefore, the number of patients decreased with time as follows: n = 19, 14, and 11 during 16–20, 36–40, and 56–60 s of PSVT, respectively, vs. n = 17, 14, and 13 during 16–20, 36–40, and 56–60 s of VT, respectively. Values are presented as mean ± standard error for data visibility. Deoxy-Hb, relative concentration of deoxygenated haemoglobin; MAP, mean arterial pressure; Oxy-Hb, relative concentration of oxygenated haemoglobin; PP, pulse pressure; TOI, tissue oxygen index.

Baseline values of heart rate (HR) and MAP were not significantly different (*P* = 0.428 and *P* = 0.0951, respectively) between patients with PSVT and those with VT (Table 2). Figure 1 shows the average cardiovascular responses during 1 min of PSVT and VT, which occurred at similar rates (151 ± 26 vs. 169 ± 50 beats/min, respectively; *P* = 0.194). MAP decreased rapidly with the arrhythmias, and the initial change in MAP tended to be greater (*P* = 0.0516) during VT (-42 ± 23 mmHg) than during PSVT (-25 ± 27 mmHg). The decrease in MAP disappeared by 40 s of PSVT, whereas a significant depressor response persisted during VT (Fig. 1A). MAP changes from the initial decrease were defined as MAP_recovery_. In both cases of PSVT and VT, MAP recovered from the initial nadir as indicated by the increase in MAP_recovery_ (*P* ≤ 0.011; Fig. 1B). Baseline pulse pressure (PP) was lower (*P* = 0.049) in patients with VT than in those with PSVT, and the initial rapid decrease in PP caused by VT was greater than that caused by PSVT (*P* < 0.001; Table 2). PP_recovery_ was calculated in the same way as MAP_recovery_. Although the decrease in PP was maintained during both PSVT and VT, PP_recovery_ reveals that PP recovered from the initial nadir irrespective of the type of tachyarrhythmia (*P* < 0.001; Fig. 1B). TOI and Oxy-Hb decreased (*P* ≤ 0.033), while Deoxy-Hb gradually increased (*P* < 0.001) throughout PSVT and VT. Respiratory rate was similar at baseline between patients with PSVT and those with VT (14 ± 4 vs. 16 ± 5 breaths/min, respectively; *P* = 0.239) and did not change significantly (*P* ≥ 0.854) during either type of tachyarrhythmia.

**Table 2:** Baseline and tachyarrhythmia-induced initial nadir or peak values of cardiovascular variables.

	PSVT (n = 19)		VT (n = 17)	
	Baseline value	Initial value	Baseline value	Initial value
HR (beats/min)	81 ± 18	151 ± 26	79 ± 22	169 ± 50
MAP (mmHg)	109 ± 21	84 ± 31	97 ± 23	55 ± 19
PP (mmHg)	80 ± 30	34 ± 18	64 ± 17*	15 ± 10*

The initial heart rate (HR) response was determined when the mean arterial pressure (MAP) and pulse pressure (PP) reached the initial nadir or peak. Values are presented as mean ± SD.

*PSVT* paroxysmal supraventricular tachycardia, *VT* ventricular tachycardia.

*Significant difference in baseline or initial values (*P* < 0.05) between the PSVT and VT groups.

### Relationships between TOI and MAP responses to tachyarrhythmias

**Figure 2:**
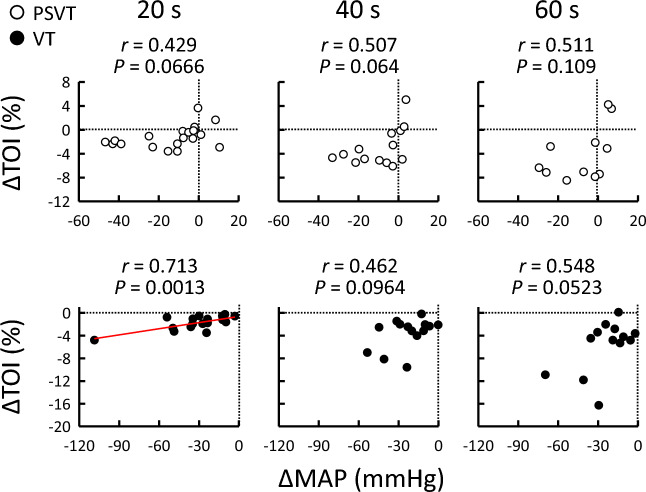
Relationship between the changes in MAP and TOI during PSVT (○) and VT (●). Vertical and horizontal dotted lines indicate zero values. The regression line (red colour) indicates when the correlation was found to be significant after Holm–Šidák correction. MAP, mean arterial pressure; PSVT, paroxysmal supraventricular tachycardia; TOI, tissue oxygen index; VT, ventricular tachycardia.

**Figure 3:**
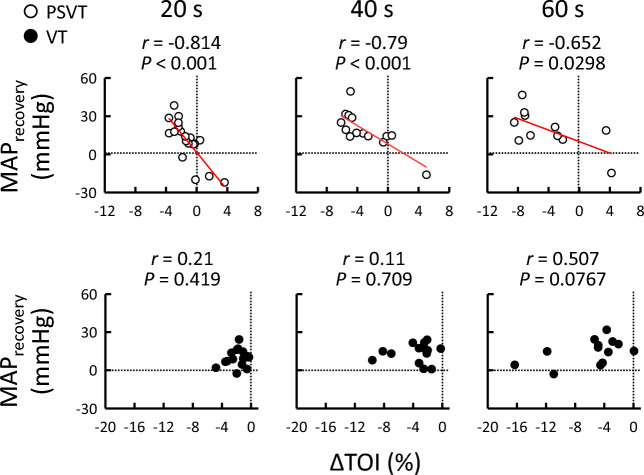
Relationship between the TOI response and MAP_recovery_ during PSVT (○) and VT (●). Vertical and horizontal dotted lines indicate zero values. The regression line (red colour) indicates when the correlation was found to be significant after Holm–Šidák correction. MAP, mean arterial pressure; PSVT, paroxysmal supraventricular tachycardia; TOI, tissue oxygen index; VT, ventricular tachycardia.

**Figure 4:**
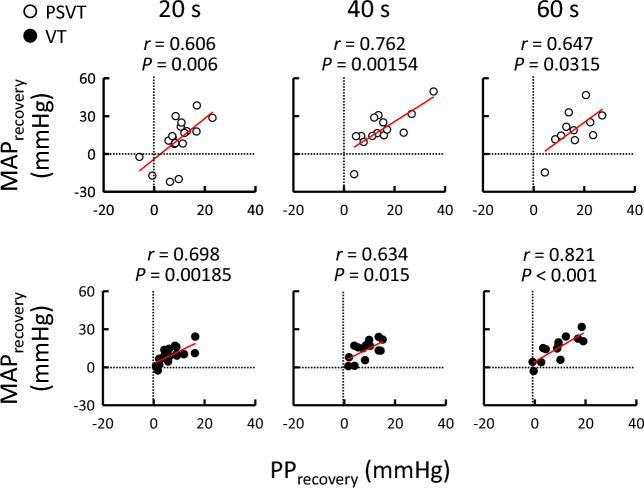
Relationship between PP and MAP recovery during PSVT (○) and VT (●). Vertical and horizontal dotted lines indicate zero values. The regression line (red colour) indicates when the correlation was found to be significant after Holm–Šidák correction. MAP, mean arterial pressure; PP, pulse pressure; PSVT, paroxysmal supraventricular tachycardia; VT, ventricular tachycardia.

The relationships between the TOI and MAP responses during PSVT and VT are shown in Figs. 2 and 3. The change in MAP from the baseline level was not significantly correlated with the TOI response (*P* ≥ 0.0523; Fig. 2) in either PSVT or VT, except at 20 s in VT. In contrast, the TOI response negatively correlated with MAP_recovery_ throughout PSVT (*r* = -0.652 to -0.814, *P* ≤ 0.0298; Fig. 3), as highlighted by the decreasing slope over time. No relationship between the TOI response and MAP_recovery_ was observed throughout VT (*P* ≥ 0.0767). Similar results were observed even after excluding data for patients taking α_1_- and β_1_-adrenergic receptor (AR) blockers, drugs infused during EPS, or those with chronic kidney disease (Table 3). The degree of initial hypotension was unrelated to the presence or absence of a significant association between the TOI response and MAP_recovery_ (Table 3). The relationship between PP and MAP recovery during PSVT and VT is summarised in Fig. 4, which illustrates whether PP recovery (attributed to SV restoration and arterial stiffening) contributes to MAP recovery during PSVT and VT in a similar manner. Interestingly, PP_recovery_ during both PSVT and VT correlated with MAP_recovery_ (*P* ≤ 0.0315).

**Table 3:** Effects of confounders on the relationship between the TOI response and MAP_recovery_.

**A. Exclusion of data of α**_**1**_**- and β**_**1**_**-adrenergic receptor blockers**			
	20 s	40 s	60 s
PSVT (n = 10–18)	− 0.814 (< 0.001)*	− 0.792 (0.00124)*	− 0.648 (0.0426)*
VT (n = 6–9)	0.458 (0.215)	0.773 (0.0715)	0.75 (0.0859)
**B. Exclusion of data of drugs infused during the EPS**			
	20 s	40 s	60 s
PSVT (n = 10–17)	− 0.732 (< 0.001)*	− 0.777 (0.00177)*	− 0.632 (0.0497)*
VT (n = 11–16)	0.296 (0.266)	0.105 (0.732)	0.258 (0.418)
**C. Exclusion of data of chronic kidney disease**			
	20 s	40 s	60 s
PSVT (n = 11–19)	− 0.814 (< 0.001)*	− 0.79 (< 0.001)*	− 0.652 (0.0298)*
VT (n = 10–13)	0.214 (0.483)	− 0.103 (0.764)	0.0717 (0.844)
**D. Effect of the initial decrease in MAP**			
	20 s	40 s	60 s
< average (n = 6–8 in PSVT; n = 6–10 in VT)	− 0.00291 (0.991)	− 0.154 (0.584)	0.169 (0.563)
> average (n = 5–11 in PSVT; n = 5–7 in VT)	− 0.677 (0.00201)*	− 0.504 (0.0788)	− 0.0638 (0.861)

Average refers to the average initial decrease in mean arterial pressure (MAP) during both paroxysmal supraventricular tachycardia (PSVT) and ventricular tachycardia (VT) (− 33 mmHg). As patients with PSVT did not have chronic kidney disease, the values were the same as those presented in Fig. 3

Values are presented as correlation coefficient (*P*-value).

*EPS* electrophysiological study, *TOI* tissue oxygen index.

*Significant correlation after Holm–Šídák correction.

## Discussion

The present study examined whether the forearm oxygenation response correlated with MAP recovery during PSVT and VT. The major findings of this study are that (1) both PSVT and VT resulted in a decrease in forearm oxygenation irrespective of MAP changes; (2) MAP recovery during PSVT was correlated with the decrease in oxygenation; (3) MAP recovery during VT was not correlated with the decrease in oxygenation, irrespective of the presence or absence of α_1_- and β_1_-AR blockers or the drugs infused during EPS; (4) the recovery of PP was related to MAP recovery during both PSVT and VT. These novel findings suggest that the decrease in forearm oxygenation, presumably caused by vasoconstriction, contributes to MAP recovery during PSVT, but is insufficient to recover MAP during VT. In contrast, PP recovery may reflect partial recovery of SV and sympathetic arterial stiffening, which subsequently assists in MAP recovery during PSVT and VT.

We observed a gradual decrease in forearm oxygenation during both PSVT and VT (Fig. 1), which indicates a reduction in peripheral tissue blood flow^16–19^. Moreover, it was likely caused by vasoconstriction rather than a decrease in perfusion pressure (i.e., MAP; Fig. 2), which is consistent with a previous study whereby ventricular pacing resulted in a decrease in forearm blood flow and an increase in vascular resistance^21^. Vasoconstriction during tachyarrhythmia is caused primarily by the arterial baroreflex to recover MAP^1–3^. Notably, the decrease in forearm oxygenation was maintained even when the MAP returned to the baseline level during PSVT (Fig. 1). This phenomenon may be due to the decrease in PP, which is another stimulus to the arterial baroreceptors^22^. Indeed, increased muscle sympathetic nerve activity appears to be maintained throughout ventricular pacing, irrespective of the recovery of MAP to pre-pacing levels^3,4^.

Decreased peripheral blood flow caused by vasoconstriction is believed to contribute to MAP recovery during tachyarrhythmia. We confirmed that the decrease in forearm oxygenation correlated negatively with the recovery of MAP throughout PSVT (Fig. 3), which indicates that as the forearm oxygenation decreased, the MAP increased. Interestingly, the effectiveness of forearm oxygenation reduction lessened with MAP restoration over time because MAP_recovery_ reached a plateau (Fig. 1), and the slope of the correlation appeared to reduce as time passed from the onset of PSVT (Fig. 3). In contrast, we found that the decrease in forearm oxygenation during VT increased with time, but was unrelated to MAP recovery (Figs. 2 and 3), suggesting that peripheral vasoconstriction did occur (preventing further hypotension), but it lacked efficacy in terms of MAP recovery. Therefore, we inferred that the reduction in peripheral blood flow caused by vasoconstriction was useful in the recovery of MAP during PSVT, but not during VT.

The differential effects of forearm oxygenation reduction on MAP recovery between the PSVT and VT groups were attributed to the variations in CO and/or the ability to evoke vasoconstriction. Previous studies have demonstrated lower SV and CO during ventricular pacing than those during atrial pacing and dual-chamber cardiac pacing at the same rate^5–8^. The same applies to the comparison between VT and PSVT. Additionally, the heart dysfunction observed in patients with VT (Table 1) may have also contributed to a decrease in CO during tachyarrhythmia, resulting in less blood delivered to the peripheral vasculature during VT. When the blood supply is lowered beyond a certain threshold, peripheral vasoconstriction cannot effectively increase peripheral vascular resistance, resulting in the failure of AP recovery. Although we were unable to measure CO in this study, our results indicate that CO during tachyarrhythmia may be a determinant of the effectiveness of peripheral vasoconstriction in MAP recovery.

Interestingly, MAP recovery correlated with PP recovery during both PSVT and VT (Fig. 4), while MAP recovery correlated with only the TOI response in PSVT (Fig. 3). Partial recovery of SV during PSVT and VT may contribute to PP and MAP recovery. In a previous study^5^, CO transiently decreased and then returned to its control level during constant atrial or ventricular pacing, presumably due to the increase in venous pressure (i.e., higher filling pressure) that resulted from the accumulation of blood in the large veins and atria. Another contributor to PP and MAP recovery is an increase in the sympathetic vasomotor outflow to the central and peripheral arteries during PSVT and VT. The augmented sympathetic vasomotor drive causes not only peripheral vasoconstriction but also arterial stiffening^23^, which lead to a larger forward wave amplitude, earlier reflected wave arrival, and greater PP^24^.

Impaired vasoconstriction may also explain the lack of relationship between decreased forearm oxygenation and MAP recovery during VT. However, the present study revealed that the reduction in forearm oxygenation during VT was independent of changes in MAP and appeared to be greater than that during PSVT (Figs. 1 and 2). These results suggest that the decrease in blood flow during VT was not simply secondary to hypotension, but was influenced by similar or greater vasoconstriction compared to that during PSVT. This inference is supported by previous findings^25–27^ that large AP changes (> 30 mmHg) evoked similar arterial baroreflex responses of sympathetic nerve activity between patients with and without left ventricular dysfunction and/or heart failure, which are common in patients with VT (Table 1)^9^. Similarly, in the present study, the change in AP (> 30 mmHg) and average depressor response during VT were equal (Fig. 2). These results suggest that sympathetic vasomotor outflow may increase to a similar or greater degree during VT than PSVT, irrespective of heart dysfunction. Such a rise in sympathetic vasomotor outflow would cause vasoconstriction at least in the forearm muscles because intraarterial (radial artery) administration of α_1_- or α_2_-adrenergic receptor (AR) agonist produced equivalent forearm vasoconstriction in normal volunteers and patients with heart failure^28^. However, caution is needed because the findings for α_1_-AR responsiveness in congestive heart failure are not unanimous^29^. Even if the α_1_-AR responsiveness was impaired by ~ 50% as shown in the common femoral artery^30^, the weak vasoconstriction would restore MAP somewhat, which might result in “gentle” slope of the relationship between decreased forearm oxygenation and MAP recovery during VT. The above background implies a low possibility that impaired vasoconstriction in patients with VT results in a lack of correlation between forearm oxygenation reduction and AP recovery; however, this should be verified by future studies.

Other possible confounders could explain the lack of a relationship between the TOI response and MAP_recovery_ during VT. First, vasoactive drugs taken before EPS, especially α_1_-AR blocker, may have reduced the ability to evoke peripheral vasoconstriction. However, excluding the data of (1) patients receiving α_1_- and β_1_-AR blockers and (2) the drugs infused during EPS had no effect on the ΔTOI-MAP_recovery_ relationship as shown in Table 3. Second, patients with VT had a higher prevalence of chronic kidney disease than those with PSVT (Table 1). However, previous studies^31,32^ have found similar baroreflex control of sympathetic activity between control participants and patients with chronic renal failure. We confirmed the lack of relationship during VT by excluding data of chronic kidney disease (Table 3). Finally, the initial decrease in MAP tended to be greater during VT than PSVT; however, the degree of initial hypotension was unrelated to the lack of the ΔTOI-MAP_recovery_ relationship (Table 3). Although we attempted to account for the confounders in the present study, other possible confounders (e.g., structural heart disease and heart dysfunction) may explain the lack of ΔTOI-MAP_recovery_ relationship during VT. Thus, our results can be considered pilot data.

The AP response and tachyarrhythmia symptoms vary greatly among patients^33^. Our findings suggest that AP recovery during tachyarrhythmia is dependent on the type of tachyarrhythmia. Patients with VT and smaller CO, as well as an impaired ability for peripheral vasoconstriction, are encouraged to receive therapy (e.g., ablation) due to poor tachyarrhythmia tolerance. Likewise, the same may be true in patients with PSVT as syncope can occur during PSVT^34,35^. If VT/PSVT patients have an impaired ability for peripheral vasoconstriction as observed in heart failure patients, chronic use of carvediol (mixed β and α_1_-AR antagonist) may improve the vascular α_1_-AR signal transduction and hence the vasoconstrictor ability with a decrease in resting AP^36^.

The present study had several limitations. First, peripheral blood flow was not directly measured, but was instead estimated according to the forearm oxygenation response. Second, the rate, duration, and AP responses during tachyarrhythmia were not artificially regulated because this study was conducted during actual tachyarrhythmias and not during cardiac pacing, to understand the clinical status of the patients. Third, it was difficult to measure CO, SV, and intraventricular pressure during tachyarrhythmia in patients because of the nature of the observational study design as well as the spontaneous nature of tachyarrhythmias, their immediate termination, and the limited time for evaluation.

In conclusion, the present study demonstrated for the first time a differential relationship between forearm oxygenation reduction, presumably caused by vasoconstriction, and MAP recovery during PSVT and VT after the exclusion of some confounders. Therefore, in clinical and daily situations, restricting peripheral blood flow is likely associated with MAP recovery during PSVT, but not during VT. This discrepancy may be the result of a difference in CO and/or the vasoconstriction ability during PSVT and VT. Predicting CO and vasoconstriction ability during tachyarrhythmia could be valuable for preventing syncope onset in clinical and everyday settings and selecting therapy.

## Methods

### Study design and patients

This was a cross-sectional observational study. This study conformed to the standard set by the Declaration of Helsinki, except for registration in a database, and the Japanese Ethical Guidelines for Medical and Health Research Involving Human Subjects. The study protocol was approved by the Institutional Review Boards of the University of Tsukuba Hospital (reference number: H28-275) and National Institute of Advanced Industrial Science and Technology (reference number: hi2018-0235).

Eligible patients were 20 years of age or older at consent and were scheduled for EPS to examine/treat PSVT and VT from April 2017 to March 2019 at the University of Tsukuba Hospital. The patients were recruited in consultation and underwent EPS at the Hospital. Written informed consent was obtained from all patients.

### Measurements during electrophysiological study

The EPS was performed with patients in a fasting state. For those who regularly took vasoactive drugs, the EPS was conducted after they had taken the drugs (Table 1). No sedatives were administered. After administering local anaesthesia to patients, a catheter was inserted into the right femoral or radial artery to measure AP. Diagnostic catheters were then inserted into the right atrial appendage, right ventricular apex, and His bundle under fluoroscopic guidance. Additional catheters were used for specific procedures in individual patients when required. AP, surface ECG, and intracardiac electrograms were recorded using a multichannel recording system (CardioLab System; Prucka Engineering, Houston, TX, USA). HR was measured using surface ECG. The exhaled breath temperature was evaluated to monitor respiratory rate which was not measured in seven (PSVT, n = 4; VT, n = 3) out of the 36 patients owing to malfunction of the instruments. The forearm oxygenation status was monitored using NIRS (NIRO-200NX, Hamamatsu Photonics, Hamamatsu, Japan). Photoemission and detection probes were placed on the patients’ skin over the left extensor carpi radialis muscle, with an interprobe distance of 4 cm. The basic principle of NIRS has been described elsewhere^37,38^.

After the above instrumentation, intracardiac stimulation was performed using an electrostimulator (SEC-5104; Nihon Kohden Corporation, Tokyo, Japan). PSVT and VT were induced using overdrive atrial or ventricular pacing, programmed atrial or ventricular stimulation, or no stimulation. Once PSVT or VT was evoked, programmed stimulation was applied to diagnose and terminate tachyarrhythmia, as necessary. Tachyarrhythmia recordings to fulfil the criteria (56 PSVTs and 73 VTs) were included in the data analysis. The following tachyarrhythmias were evoked under drug infusion: 13 PSVTs and 18 VTs following isoproterenol infusion, 4 VTs following atropine infusion, and 13 VTs following thiamylal sodium infusion. After or before EPS, heart function and structure were assessed using ultrasonic echocardiography.

### Data treatments and statistical analyses

Surface ECG, AP, exhaled breath temperature, and NIRS-derived variables were measured throughout the EPS and stored on a computer at a sampling frequency of 1,000 Hz (PowerLab 16/35, ADInstruments-Japan, Nagoya, Japan). MAP and PP were calculated for each cardiac cycle. Normality and equal variance tests (Shapiro–Wilk test and Brown-Forsythe test, respectively) were conducted before selecting the method of statistical analyses, and a one-way repeated measures ANOVA was used only once these tests were passed. Since the number of patients was decreased with time, one-way repeated measures ANOVA was used with a general linear model to compute the expected mean squares.

All data were aligned at the onset of arrhythmia and sequentially averaged every 1 s. Baseline values were defined as the average values over the period before the onset of arrhythmia. Subsequently, the data for multiple PSVTs or VTs were averaged for each patient. Baseline values were compared between the PSVT and VT groups using the Mann–Whitney U test, Student’s *t*-test, or Welch’s t-test. The changes in the variables from baseline levels were analysed in three time periods (16–20, 36–40, and 56–60 s of arrhythmia) using an appropriate one-way ANOVA and post hoc test (Holm-Šídák or Dunn).

The initial nadir or peak MAP and PP values during tachyarrhythmia were defined as MAP_nadir/peak_ and PP_nadir/peak_, respectively. The degrees of MAP and PP recovery from the initial responses were calculated in the three time periods as the average changes in MAP or PP from MAP_nadir/peak_ or PP_nadir/peak_ and were defined as MAP_recovery_ and PP_recovery_, respectively. Significant changes of the MAP_recovery_ and PP_recovery_ (i.e., MAP and PP recovery from the initial nadir or peak) were analysed by the appropriate one-way ANOVA and post hoc test. To test the hypothesis that peripheral blood flow responses contribute to the MAP response during PSVT but not during VT, the relationship between forearm oxygenation and AP responses was examined during the three time periods using Pearson’s correlation analysis with post hoc Holm–Šídák for multiple comparisons. Then, association analysis was performed after excluding the data of some confounding variables. The relationship between PP_recovery_ and MAP_recovery_ was similarly analysed to examine whether PP recovery, which depends on SV restoration and arterial stiffening, contributes to MAP recovery, irrespective of PSVT and VT. To examine the influence of initial hypotension on the relationship between the forearm oxygenation and AP responses, correlation analysis was performed after the data were stratified into two groups based on the average initial hypotension during both tachyarrhythmias (above and below). Clinical characteristics were compared between the PSVT and VT groups using the Student’s *t*-test or Fisher’s exact test.

All variables were expressed as the mean ± SD, unless otherwise specified. All statistical analyses were performed using SigmaPlot® version 12.5 (Systat Software, San Jose, CA, USA). Statistical significance was defined as *P* < 0.05.

## Data Availability

The data that support the findings of this study are available from the Automotive and Medical Concert Consortium, but restrictions apply to the availability of these data, which were used under license for the current study, and so are not publicly available. Data are however available from the corresponding author upon reasonable request and with permission of the Automotive and Medical Concert Consortium.
